# The Throat and Voice. American Health Primer

**Published:** 1880-01

**Authors:** 


*The Throat and the Voice.* By J. Solis Cohen, M. D. Publishers, Lindsay & Blackiston, Philadelphia.

This is another of the series of American Health Primers, and like its companions is a useful and instructive treatise. Commencing with the general construction of the throat, its diseases are noticed in an interesting and comprehensive manner. Part second relates to the voice, commencing with the acoustics, varieties, culture, improper use of, defects, vocal gymnastics, and ending with the care of the voice.

Like the other volumes of the series, this treatise is illustrated by engravings which assist in understanding the text relating to the anatomy of the throat, and vocal apparatus.

